# Supplementary material for: Impact of urban structure on infectious disease spreading
Source: Sci Rep. 2022 Mar 9;12:3816. doi: 10.1038/s41598-022-06720-8 (PMC8907266; doi:10.1038/s41598-022-06720-8)
Supplement: Supplementary file 1 — Supplementary Information 1. [file 41598_2022_6720_MOESM1_ESM.pdf]

Supplementary Information
Impact of urban structure on infectious disease spreading

J. Aguilar, A. Bassolas, G. Ghoshal, S. Hazarie, A. Kirkley, M. Mazzoli, S. Meloni, S. Mimar, V. Nicosia, J.J. Ramasco & A. Sadilek

Table of Contents

1 Response to the lockdown **2**

2 Calculation of Transfer Entropy **6**

3 Flow hierarchy **7**
3.1 Values of Φ per city 7
3.2 Tables of counties 7

4 Measure of M from zip code LODES commuting data **11**

5 Exploring the parameters of the model and the robustness of the results **12**
5.1 Varying the infectivity β, lockdown onset π_{th} and the fraction of population that leaves residence area $\mathbf{M}$ 12
5.2 Varying the mobility input data: The model with commuting 16
5.3 Model with age structure in Paris and London 18
5.4 Model with heterogeneous X_S 22
5.5 Model synchronization of mobility reduction and epidemics 24

6 Further insights on the available COVID data and parameters estimation **25**

7 Variables and parameters **29**

8 Implementation of recurrent mobility in the model **30**

1 Response to the lockdown

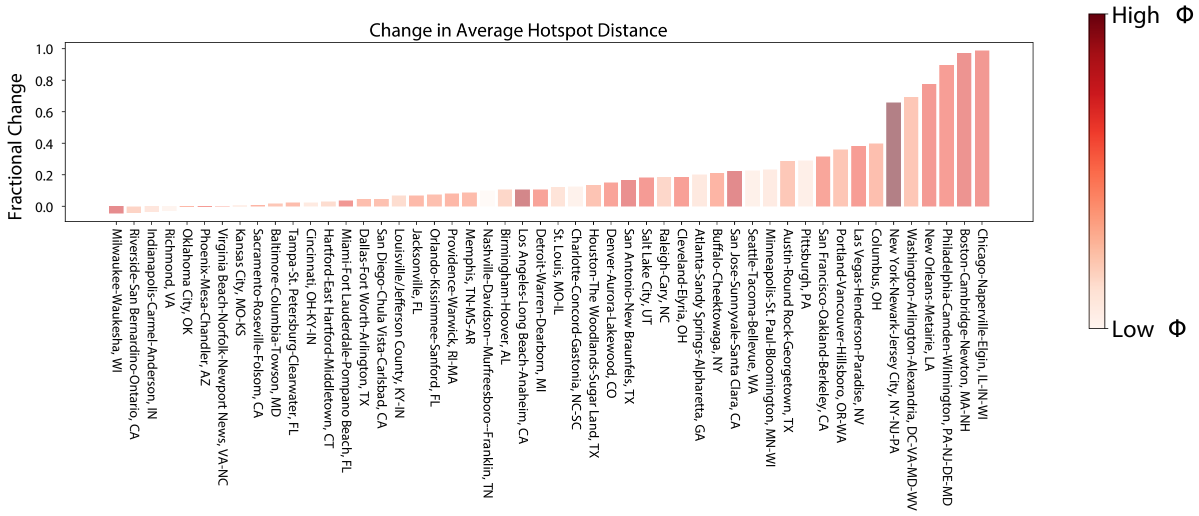

Figure S1: **Distances between hotspots**. Fractional change in average hotspot distance from the week of January 5 to the week of June 7 for the top 50 CBSAs in the US, colored by the hierarchy as of January 5.

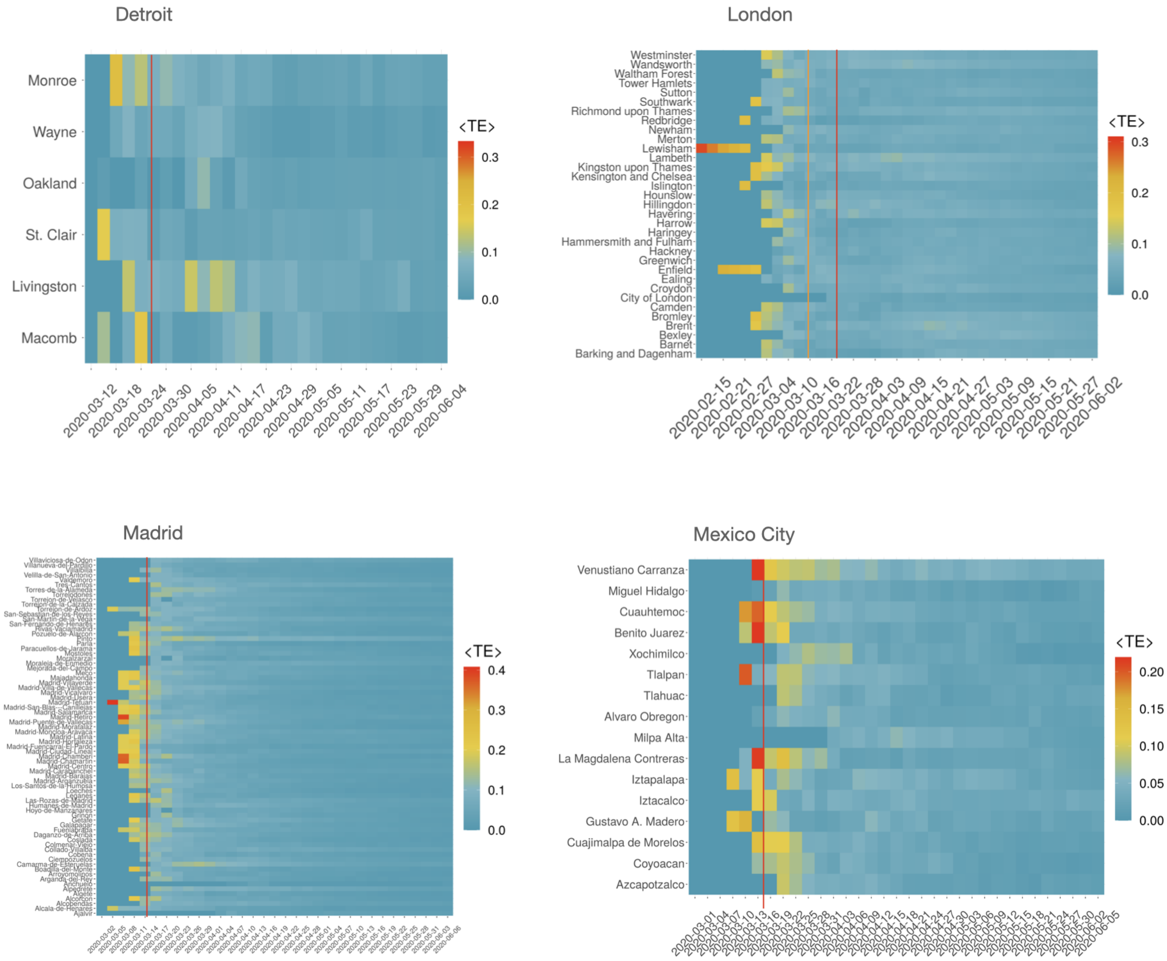

Figure S2: **Average Transfer Entropy ⟨TE⟩ for each administrative division (county or borough) with respect to the others as a function of time.** Shown are six different cities: Detroit, London, Madrid and Mexico city. Vertical red lines mark the date of the official lockdown. For London the orange line marks an advisory from the Prime Minister's office to suspend all non essential activities, which occurred one week before the lockdown (red line).

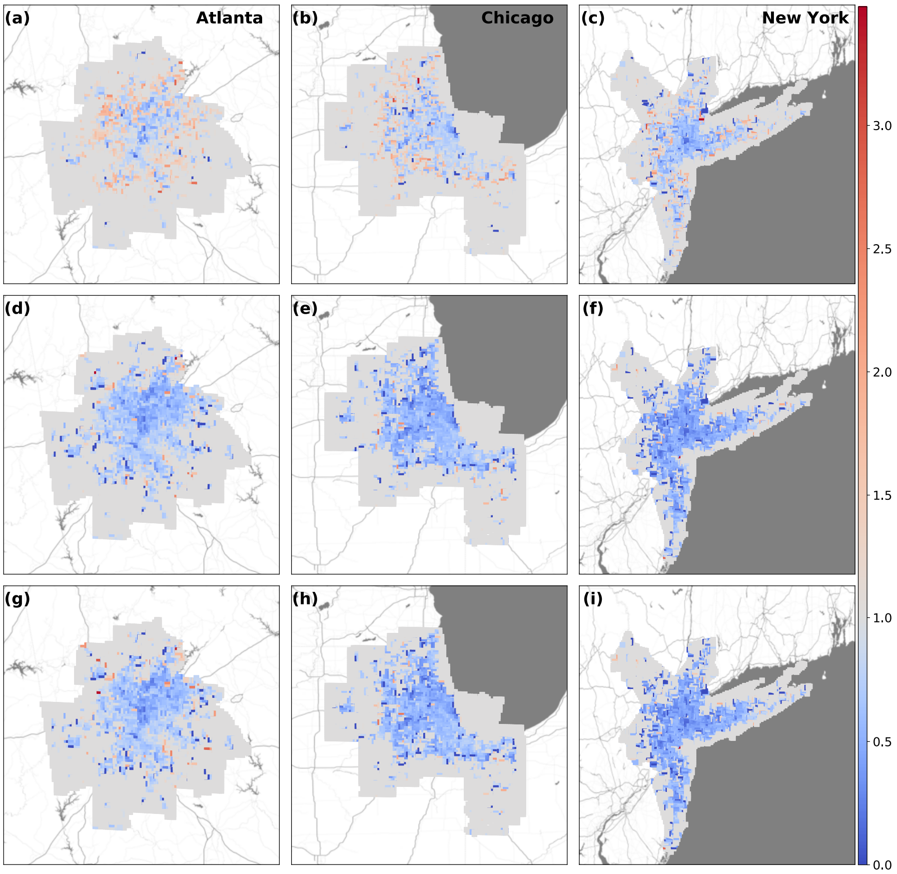

Figure S3: **Localization of flow in three cities with different mobility hierarchy.** Flow distributions in the month of April in Atlanta, Chicago and New York are compared to the baseline mobility level in February. Cells with no flow change are colored grey and have mobility ratio 1. Mobility increase and decrease are illustrated by red and blue colors for cells with mobility ratio greater than and less than 1, respectively. Panels **a, b, c** represent self-flows, **d, e, f** represent in-flows, and **g, h, i** represent out-flows.

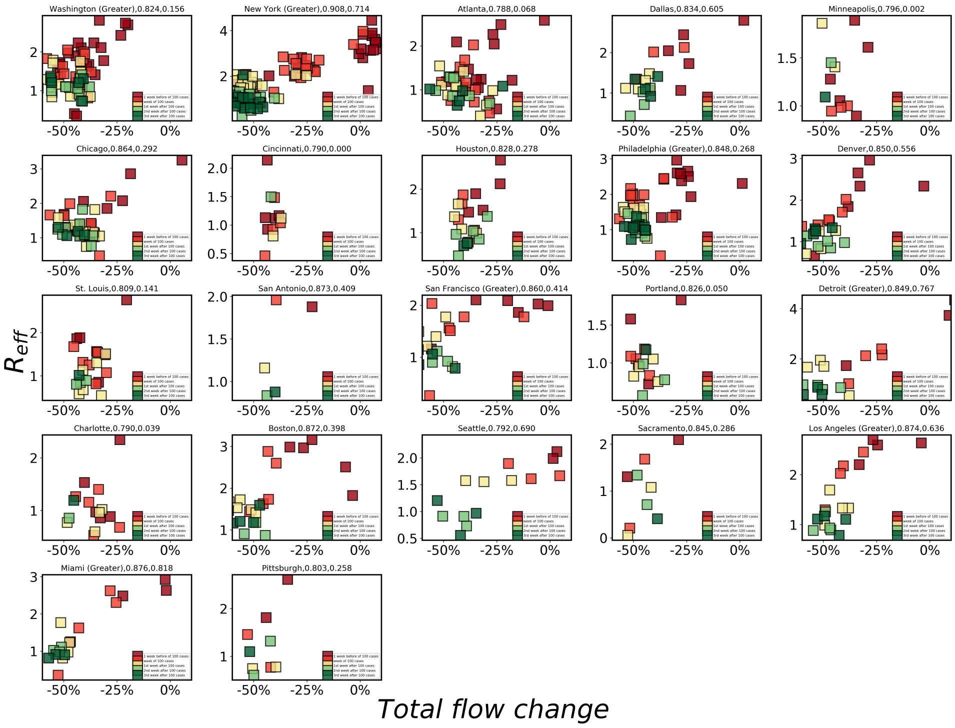

Figure S4: **Reduction of $R_{\rm eff}$ versus relative reduction of total flow at the county level in USA Metro Areas.** $R_{\rm eff}$ versus total flow reduction in counties within the same city, measured from one week before the onset to three weeks after the onset. $R_{\rm eff}$ is taken with one week of delay with respect to mobility data. This data is used to measure the correlations shown in Fig. 3 of the main paper. Only counties with more than 100 accumulated cases in the full observation period are shown.

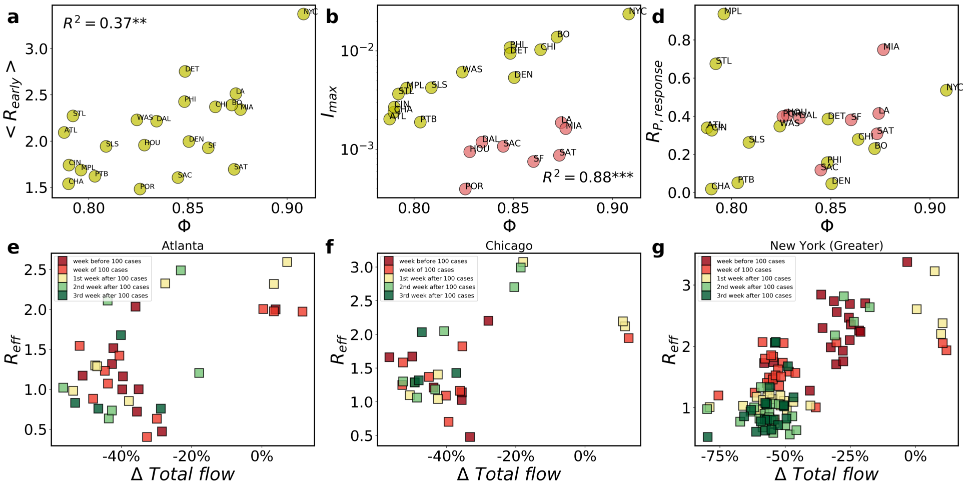

Figure S5: **Reproduction of Fig. 3 with alternative data.** The same panels of the Fig. 3 of the main paper with data from USAFacts. **a** Average R_{eff} over three weeks after the onset of 100 cases as a function of Φ. Initial transmission increases with centralization. **b** Maximum incidence $I_{\max}$ (infections per capita). Cities in pale yellow have already peaked, while infections continue to grow in those marked in red. The figure suggests the extent of spread is strongly correlated with centralization. In **d**, synchronization of mobility reduction and contagion spread among city counties measured through the Pearson coefficient of plots as those in panels **e-g**, which reproduce those of shown in Fig. 2 **g-i** of the main manuscript for Atlanta, Chicago and NYC. The panel Fig. 3**c** is not reproduced because it is only based on mobility data and it does not change with the source of the COVID-19 case information.

2 Calculation of Transfer Entropy

In order to quantify the driving in the spreading between different areas of a city, we calculated the transfer entropy [1] between the time series of the disease incidence at the county level for time windows of increasing sizes. Starting from the first reported case to the latest available data (in most cases, the first week of June 2020). For each time window, the driving each administrative unit i had over the others is calculated as the average transfer entropy $\langle \text{TE} \rangle_i = 1/N \sum_j \text{TE}_{ij}$ between the incidence time series of unit i and all the other units j, with N the number of administrative units in the city. The transfer entropy was evaluated using the *RTransferEntropy* library (`https://cran.r-project.org/web/packages/RTransferEntropy/vignettes/transfer-entropy.html`) in R, that also provides an estimation of the statistical significance of the results.

3 Flow hierarchy

3.1 Values of Φ per city

US cities show a wide spectrum of values of Φ. It goes from hierarchical (centralized) urban areas as New York City to sprawled (extended) ones as Atlanta. Table S3.1 contains a list of the 22 metropolitan areas considered in Fig. 3 and their corresponding Φ values.

Table S1: **Table of US metro areas (and their short names) considered in Figure 2 as function of Φ.**

Metro Area	Shortening	Φ	Metro Area	Shortening	Φ
New York (Greater)	NYC	0.908	Dallas	DAL	0.834
Miami (Greater)	MIA	0.876	Houston	HOU	0.828
Los Angeles (Greater)	LA	0.874	Portland	POR	0.826
San Antonio	SAT	0.873	Washington (Greater)	WAS	0.824
Boston	BO	0.872	St. Louis	SLS	0.809
Chicago	CHI	0.864	Pittsburgh	PTB	0.803
San Francisco (Greater)	SF	0.860	Minneapolis	MPL	0.796
Denver	DEN	0.850	Seattle	STL	0.792
Detroit (Greater)	DET	0.849	Cincinnati	CIN	0.790
Philadelphia (Greater)	PHI	0.848	Charlotte	CHA	0.790
Sacramento	SAC	0.845	Atlanta	ATL	0.788

3.2 Tables of counties

Table S2: **Table of counties per OCDE metropolitan area for New York City.**

County	County	County	County
Bergen County	Bronx County	Essex County	Hudson County
Hunterdon County	Kings County	Middlesex County	Monmouth County
Monroe County	Morris County	Nassau County	New York County
Ocean County	Orange County	Passaic County	Pike County
Putnam County	Queens County	Richmond County	Rockland County
Somerset County	Suffolk County	Sussex County	Union County
Warren County	Westchester County		

Table S3: **Table of counties per OCDE metropolitan area for Miami.**

County	County	County	County
Broward County	Martin County	Miami-Dade County	Palm Beach County

Table S4: **Table of counties per OCDE metropolitan area for Los Angeles.**

County	County	County	County
Los Angeles County	Orange County	Riverside County	San Bernardino County

Table S5: **Table of counties per OCDE metropolitan area for San Antonio.**

County	County	County	County
Atascosa County	Bandera County	Bexar County	Comal County
Frio County	Guadalupe County	Kendall County	Medina County
Wilson County			

Table S6: **Table of counties per OCDE metropolitan area for Boston.**

County	County	County	County
Essex County	Middlesex County	Norfolk County	Plymouth County
Suffolk County			

Table S7: **Table of counties per OCDE metropolitan area for Chicago.**

County	County	County	County
Cook County	DeKalb County	DuPage County	Grundy County
Jasper County	Kane County	Kendall County	Kenosha County
Lake County	McHenry County	Newton County	Porter County
Will County			

Table S8: **Table of counties per OCDE metropolitan area for San Francisco.**

County	County	County	County
Alameda County	Contra Costa County	Marin County	San Benito County
San Francisco County	San Mateo County	Santa Clara County	

Table S9: **Table of counties per OCDE metropolitan area for Denver.**

County	County	County	County
Adams County	Arapahoe County	Broomfield County	Clear Creek County
Denver County	Douglas County	Elbert County	Gilpin County
Jefferson County	Park County		

Table S10: **Table of counties per OCDE metropolitan area for Detroit.**

County	County	County	County
Livingston County	Macomb County	Monroe County	Oakland County
St. Clair County	Wayne County		

Table S11: **Table of counties per OCDE metropolitan area for Philadelphia.**

County	County	County	County
Bucks County	Burlington County	Camden County	Cecil County
Chester County	Delaware County	Gloucester County	Mercer County
Montgomery County	New Castle County	Philadelphia County	Salem County

Table S12: **Table of counties per OCDE metropolitan area for Sacramento.**

County	County	County	County
El Dorado County	Placer County	Sacramento County	Yolo County

Table S13: **Table of counties per OCDE metropolitan area for Dallas.**

County	County	County	County
Collin County	Cooke County	Dallas County	Denton County
Ellis County	Fannin County	Hood County	Hunt County
Johnson County	Kaufman County	Palo Pinto County	Parker County
Rains County	Rockwall County	Somervell County	Tarrant County
Van Zandt County	Wise County		

Table S14: **Table of counties per OCDE metropolitan area for Houston.**

County	County	County	County
Austin County	Brazoria County	Chambers County	Colorado County
Fort Bend County	Galveston County	Harris County	Liberty County
Montgomery County	Polk County	San Jacinto County	Waller County

Table S15: **Table of counties per OCDE metropolitan area for Portland.**

County	County	County	County
Clackamas County	Clark County	Columbia County	Cowlitz County
Multnomah County	Skamania County	Washington County	

Table S16: **Table of counties per OCDE metropolitan area for Washington.**

County	County	County	County
Alexandria city	Anne Arundel County	Arlington County	Baltimore County
Baltimore city	Calvert County	Carroll County	Charles County
Clarke County	Culpeper County	District of Columbia	Fairfax County
Falls Church city	Fauquier County	Frederick County	Fredericksburg city
Harford County	Howard County	Jefferson County	Loudoun County
Montgomery County	Prince George's County	Prince William County	Rappahannock County
Spotsylvania County	St. Mary's County	Stafford County	Warren County

Table S17: **Table of counties per OCDE metropolitan area for St. Louis.**

County	County	County	County
Jefferson County	Jersey County	Lincoln County	Madison County
Monroe County	St. Charles County	St. Clair County	St. Louis County
St. Louis city	Warren County		

Table S18: **Table of counties per OCDE metropolitan area for Pittsburgh.**

County	County	County	County
Allegheny County	Washington County	Westmoreland County	

Table S19: **Table of counties per OCDE metropolitan area for Minneapolis.**

County	County	County	County
Anoka County	Carver County	Chisago County	Dakota County
Hennepin County	Isanti County	Kanabec County	Pierce County
Ramsey County	Scott County	Sherburne County	St. Croix County
Washington County	Wright County		

Table S20: **Table of counties per OCDE metropolitan area for Seattle.**

County	County	County	County
King County	Pierce County	Snohomish County	Thurston County

Table S21: **Table of counties per OCDE metropolitan area for Cincinnati.**

County	County	County	County
Boone County	Bracken County	Butler County	Campbell County
Clermont County	Dearborn County	Gallatin County	Grant County
Hamilton County	Kenton County	Ohio County	Pendleton County
Warren County			

Table S22: **Table of counties per OCDE metropolitan area for Charlotte.**

County	County	County	County
Cabarrus County	Gaston County	Mecklenburg County	Union County
York County			

Table S23: **Table of counties per OCDE metropolitan area for Atlanta.**

County	County	County	County
Barrow County	Bartow County	Butts County	Cherokee County
Clayton County	Cobb County	Coweta County	Dawson County
DeKalb County	Douglas County	Fayette County	Forsyth County
Fulton County	Gwinnett County	Henry County	Newton County
Paulding County	Rockdale County	Walton County	

4 Measure of M from zip code LODES commuting data

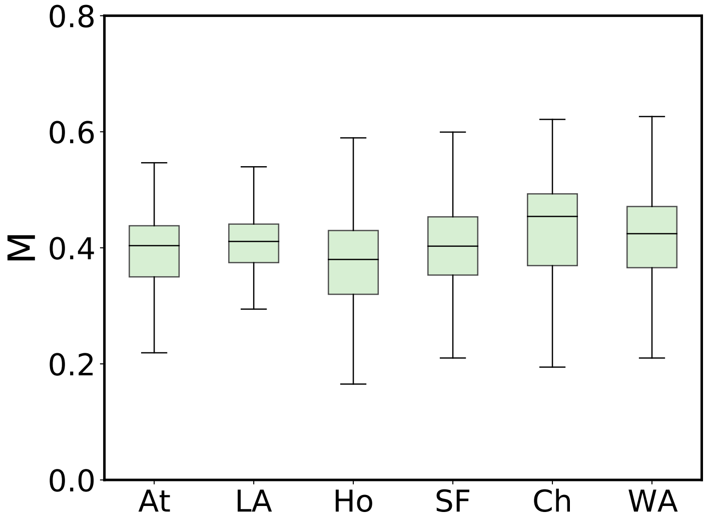

Figure S6: **Measure of M with commuting data for different US cities: At**lanta, **L**os **A**ngeles, **Ho**uston, **S**an **F**rancisco, **Ch**icago and **Wa**shington. We get an estimation of M for every zip code as the portion of population commuting outside its residence zip code. Each box reflects all the zip codes of the city and displays the median, quartiles, the 5% and 95% confidence intervals. In this way, we show evidence that justifies the $M = 0.4$ value used in the main text.

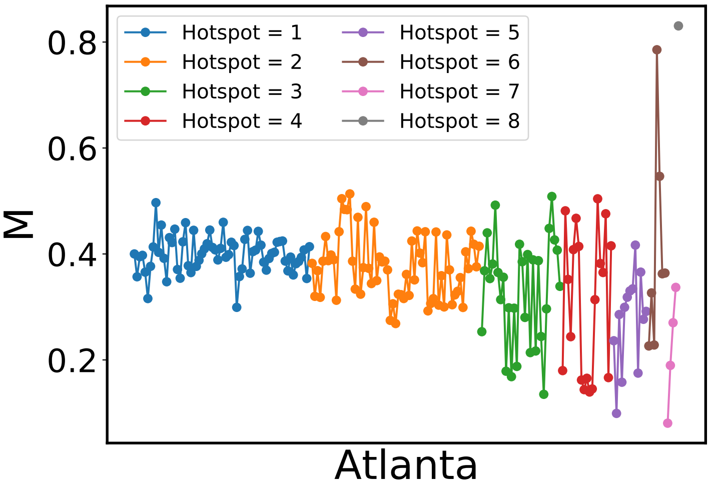

Figure S7: **Measure of M for all the zip codes in Atlanta using commuting data:**. We get an estimation of M for every zip code as the portion of population commuting outside its residence zip code. This figure complements the previous one (fig S6) as it shows how M fluctuates through the city around its mean value.The zip codes are grouped by colors representing its hotspot level, showing that the dispersion of M increase with the hotspot level.

5 Exploring the parameters of the model and the robustness of the results

To confirm the qualitative trends presented in the main text, here we systematically explore the effects of different parameters on the model results, as well as run our model on a different dataset.

5.1 Varying the infectivity β, lockdown onset $\pi_{\mathbf{th}}$ and the fraction of population that leaves residence area M

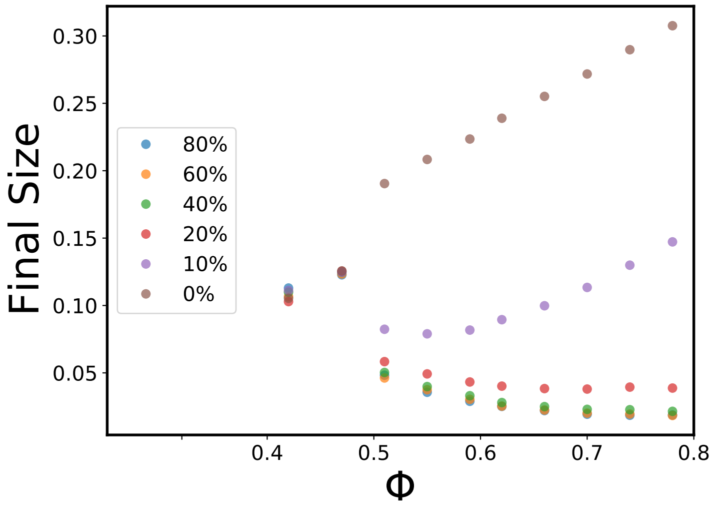

Figure S8: **Model with $\mathbf{R_0} \approx$ 1.2.** Simulations run with the same parameters as Fig. 5**a** but with $\beta = 0.31$ days^{-1} inducing a reproduction number $R_0 \approx 1.2$ for a city generated with the mobility network and total population of the Atlanta metropolitan area. The value of the epidemic size is explored as a function of Φ for different values of X_S that are shown in the legend. In this way, it is possible to observe the inversion of the size versus Φ curves for strong lockdowns.

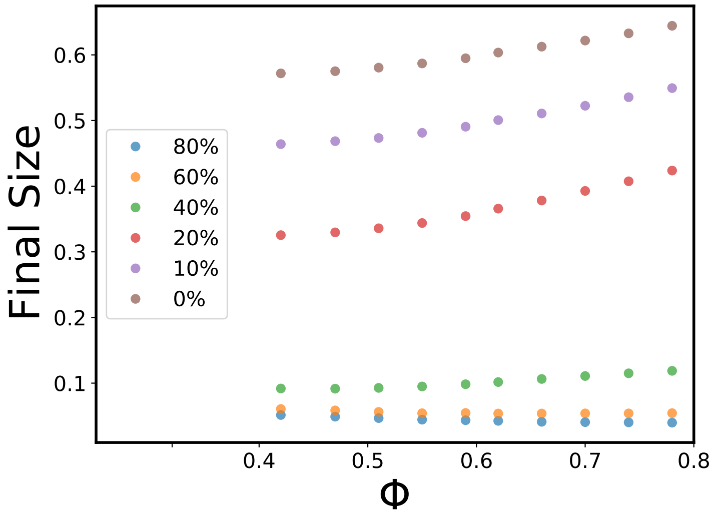

Figure S9: **Model with $\mathbf{R_0} \approx$ 1.6.** Simulations run with the same parameters as Fig. 5**a** ($\beta = 0.42$ days^{-1}) producing a reproduction number $R_0 \approx 1.6$ for a city generated with the mobility network and total population of the Atlanta metropolitan area. The value of the epidemic size is explored as a function of Φ for different values of X_S that are shown in the legend. In this way, it is possible to observe the inversion of the size versus Φ curves for strong lockdowns.

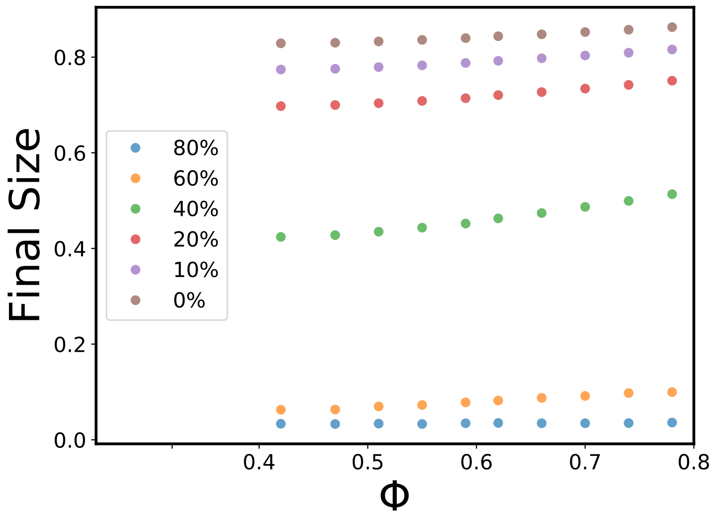

Figure S10: **Model with $\mathbf{R_0} \approx$ 2.3.** Simulations run with the same parameters as Fig. 5**a** but with $\beta = 0.6$ days^{-1} inducing a reproduction number $R_0 \approx 2.3$ for a city generated with the mobility network and total population of the Atlanta metropolitan area. The value of the epidemic size is explored as a function of Φ for different values of X_S that are shown in the legend. In this way, it is possible to observe the inversion of the size versus Φ curves for strong lockdowns.

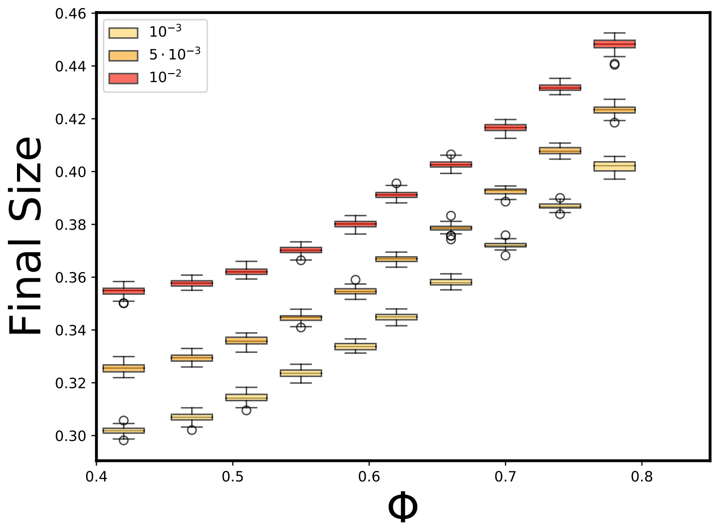

Figure S11: **Model with soft lockdown, different π_{th} values** Simulations run with the same parameters as Fig. 5**a** but with $X_S = 0.2$ for a city generated with the mobility network and total population of the Atlanta metropolitan area. The value of the epidemic size is explored as a function of Φ for different values of π_{th} that are shown in the legend. In this way, it is possible to observe that the trend showed in the Figs. 4**d**, 5**b** and 5**c** do not depend on a fine tuning of π_{th}. We then can assert that the epidemic size grows with Φ for a soft lockdown

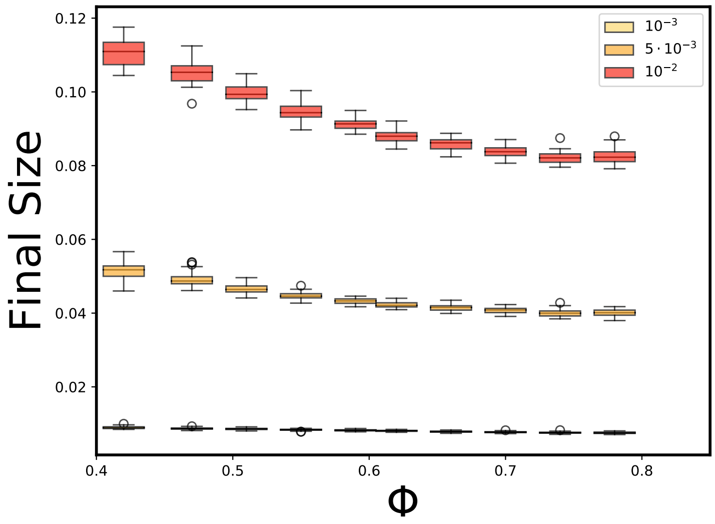

Figure S12: **Model with hard lockdown, different π_{th} values** Simulations run with the same parameters as Fig. 5**a** with $X_S = 0.8$ for a city generated with the mobility network and total population of the Atlanta metropolitan area. The value of the epidemic size is explored as a function of Φ for different values of π_{th} that are shown in the legend. In this way, it is possible to observe that a hard lockdown will either erase the Φ dependence on the epidemic size or generate the inversion (epidemic size decreases with Φ).

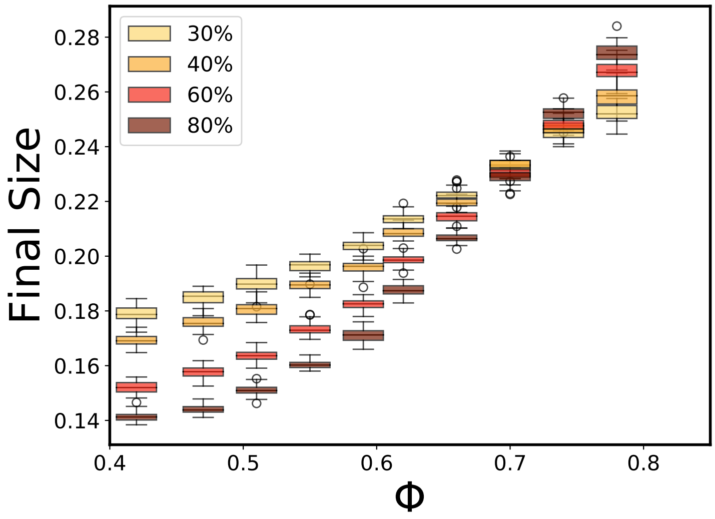

Figure S13: **Model with soft lockdown, different values for M** Simulations run with the same parameters as Fig. 5**a** but with $X_S = 0.3$ for a city generated with the mobility network and total population of the Atlanta metropolitan area. The value of the epidemic size is explored as a function of Φ for different values of M that are shown in the legend. In this way, it is possible to observe that the trend showed in the Figs 4**d**, 5**b** and 5**c** do not depend on a fine tuning of M.

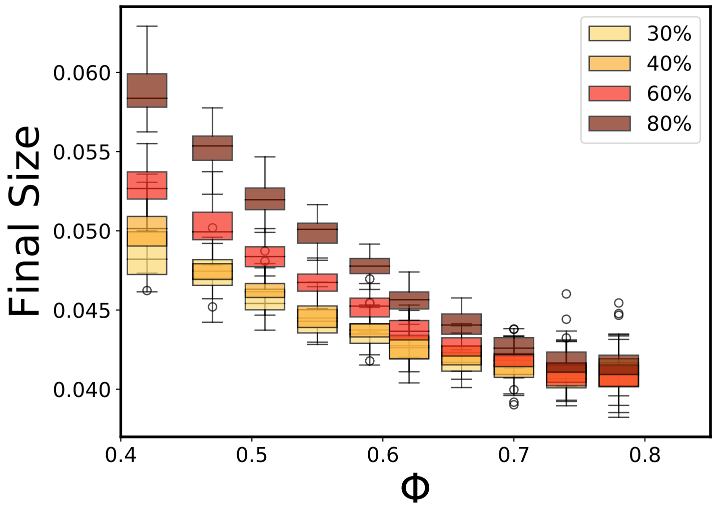

Figure S14: **Model with hard lockdown, different values for M** Simulations run with the same parameters as Fig. 5**a** with $X_S = 0.8$ for a city generated with the mobility network and total population of the Atlanta metropolitan area. The value of the epidemic size is explored as a function of Φ for different values of M that are shown in the legend. In this way, it is possible to observe that the trend showed in the Fig. 5**a** do not depend on a fine tuning of M.

5.2 Varying the mobility input data: The model with commuting

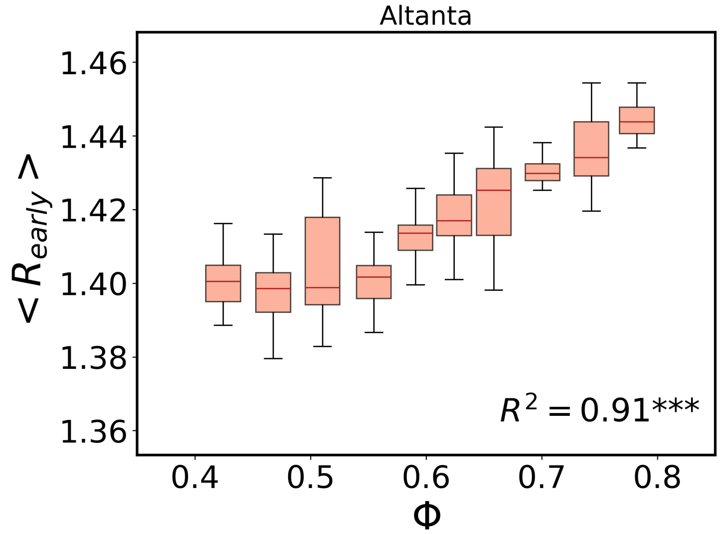

Figure S15: R_{eff} **with commuting data** Simulations run with the same parameters as Fig. 4 for a city generated with the mobility network and total population of the Atlanta metropolitan area. The value of R_{eff} in the early stages explored as a function of Φ. In this way, it is possible to observe that the trend showed in the Fig. 2**a** is independent on the type of mobility data

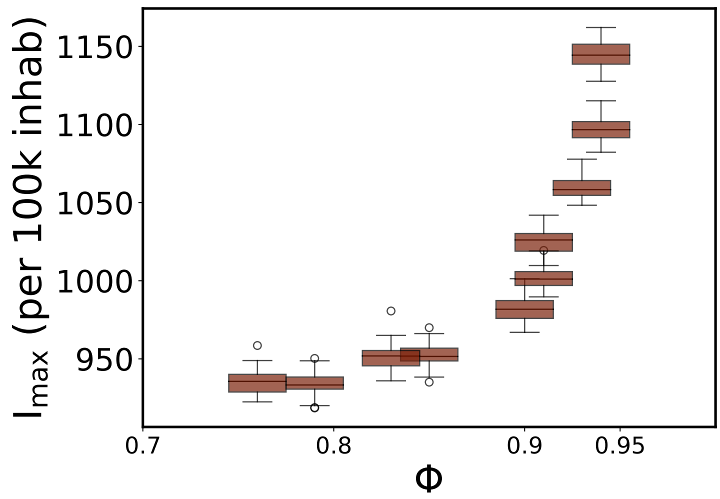

Figure S16: I_{max} **with commuting data** Simulations run with the same parameters as Fig. 4 for a city generated with the mobility network and total population of the Atlanta metropolitan area. The peak incidence I_{max} explored as a function of Φ. In this way, it is possible to observe that the trend showed in the Fig. 4**b** is independent of the mobility data

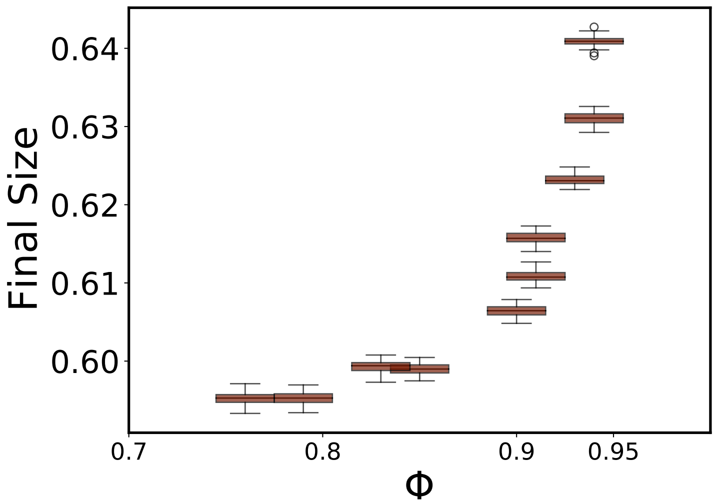

Figure S17: **Epidemic size without lockdown with commuting data** Simulations run with the same parameters as Fig. 4 for a city generated with the mobility network and total population of the Atlanta metropolitan area. The epidemic size explored as a function of Φ. In this way, it is possible to observe that the trend showed in the Fig.4**d** is independent of the mobility data

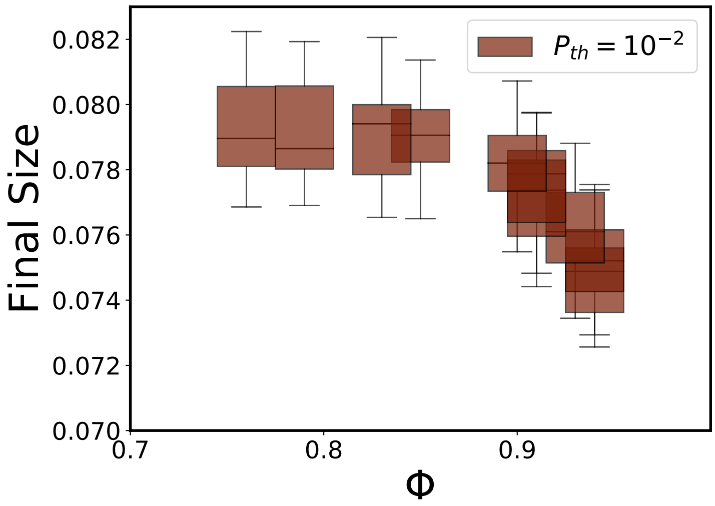

Figure S18: **Epidemic size with commuting data** Simulations run with the same parameters as Fig. 5**a** for a city generated with the mobility network and total population of the Atlanta metropolitan area. The epidemic size explored as a function of Φ. In this way, it is possible to observe that the trend showed in the Fig. 5**a** is independent of the mobility data

5.3 Model with age structure in Paris and London

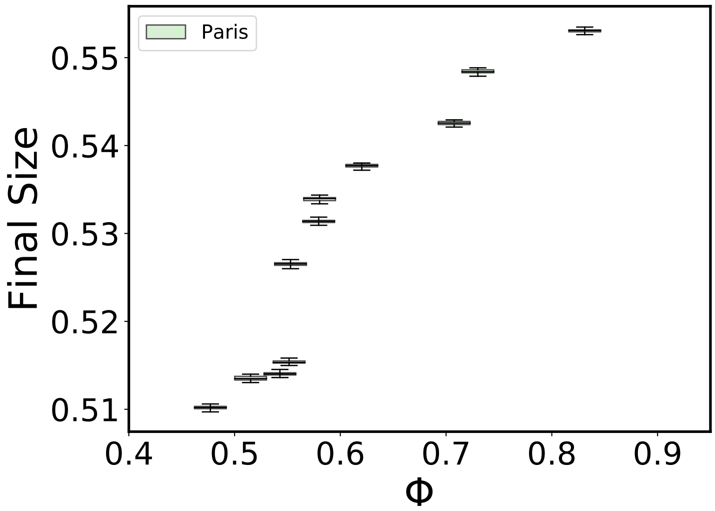

Figure S19: **Commuting data and census population: Without Lockdown** Simulations run with realistic implementation in Paris. The commuting data is diversified by two types: Students and workers that we associate to mobility in the ages [0,18] and [19,64+] respectively. Public census data grouped by age is used instead of the artificial populations proportional to flow of the main discussion. The free epidemic size is explored as a function of Φ, including just a baseline contact matrix [2] . The detailed model recovers the main result of this work: the epidemic outbreak without restrictions grows with Φ

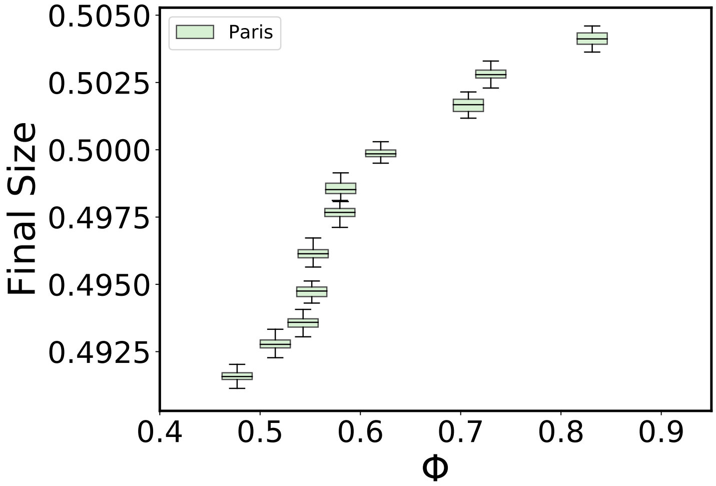

Figure S20: **Commuting data and census population: Soft lockdown** Simulations run with realistic implementation in Paris. The commuting data is diversified by two types: Students and workers that we associate to mobility in the ages [0,18] and [19,64+] respectively. Public census data grouped by age is used instead of the artificial populations proportional to flow of the main discussion. The epidemic size is explored as a function of Φ with a soft restriction scenario as described in [2] "School closure and senior isolation". The detailed model also shows that a soft set of restrictions would be less effective in centralized cities.

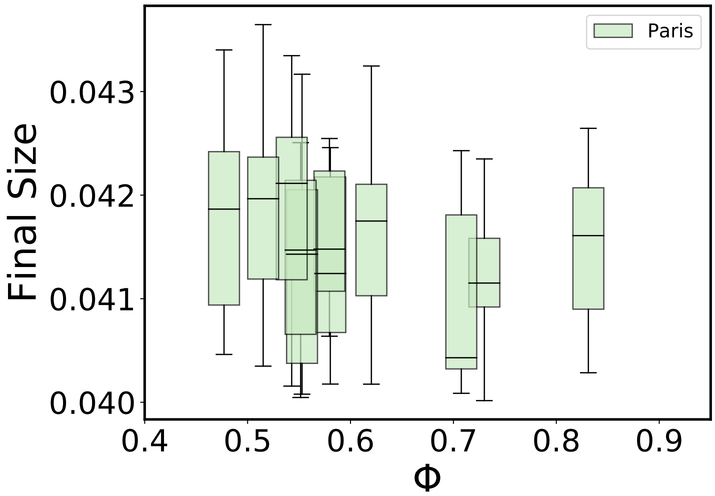

Figure S21: **Commuting data and census population: Hard lockdown** Simulations run with realistic implementation in Paris. The commuting data is diversified by two types: Students and workers that we associate to mobility in the ages [0,18] and [19,64+] respectively. Public census data grouped by age is used instead of the artificial populations proportional to flow of the main discussion. The epidemic size is explored as a function of Φ with a hard lockdown situation as described in [2] .We show that, if the mobility and social restrictions are hard enough, it is possible to erase the Φ dependence. Therefore, this crucial finding is independent of the level of detail of the model

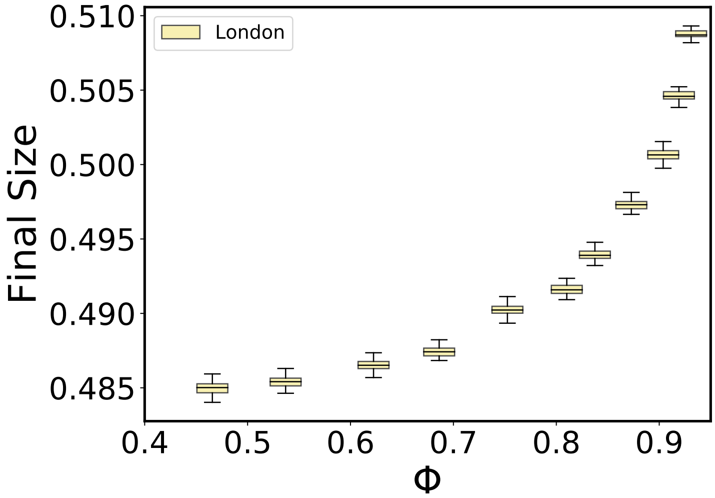

Figure S22: **Commuting data and census population: No lockdown in London** Simulations run with realistic implementation in London. Public census data grouped by age is used instead of the artificial populations proportional to flow of the main discussion. The free epidemic size is explored as a function of Φ, including just a baseline contact matrix [2] . The detailed model recovers the main result of this work: the epidemic outbreak without restrictions grows with Φ

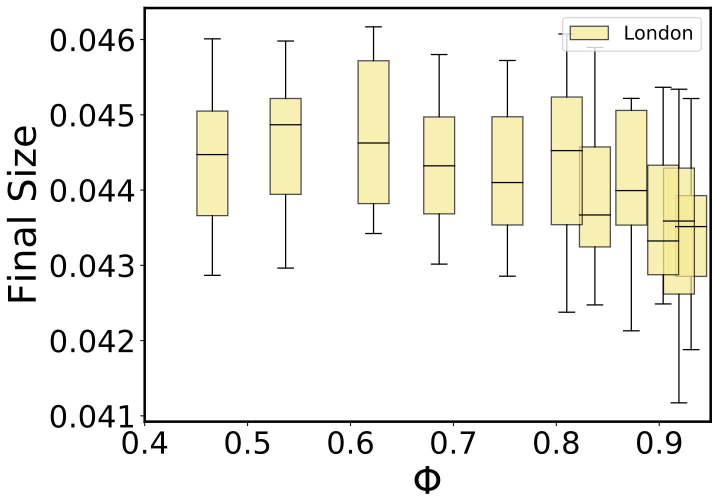

Figure S23: **Commuting data and census population: Hard lockdown in London** Simulations run with realistic implementation in London. Public census data grouped by age is used instead of the artificial populations proportional to flow of the main discussion. The epidemic size is explored as a function of Φ with a hard lockdown situation as described in [2] .We show that, if the mobility and social restrictions are hard enough, it is possible to erase the Φ dependence. Therefore, this crucial finding is independent of the level of detail of the model

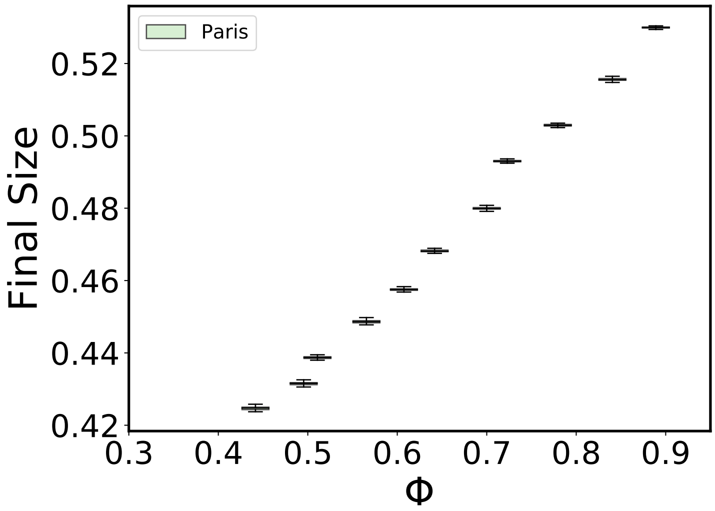

Figure S24: **Google mobility data and population according to flow: Without Lockdown** Simulations run with realistic implementation in Paris. The population is distributed proportionally to the flow of every node, as in main text. The age diversification is included following the public census data proportions. The free epidemic size is explored as a function of Φ, including just the baseline contact matrix [2] "School closure and senior isolation". The detailed model recovers the main result of this work: the epidemic outbreak without restrictions grows with Φ

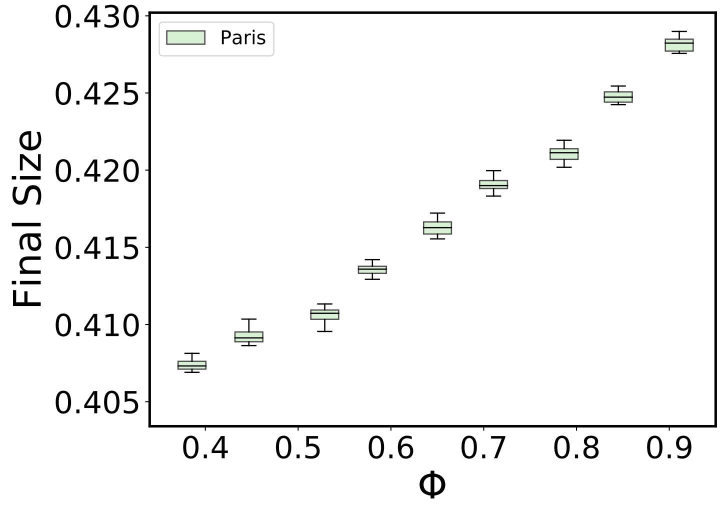

Figure S25: **Google mobility data and population according to flow: Soft lockdown** Simulations run with realistic implementation in Paris. The population is distributed proportionally to the flow of every node, as in main text. The age diversification is included following the public census data proportions. The epidemic size is explored as a function of Φ with a soft restriction scenario as described in [2] "School closure and senior isolation". The detailed model also shows that a soft set of restrictions would be less effective in centralized cities.

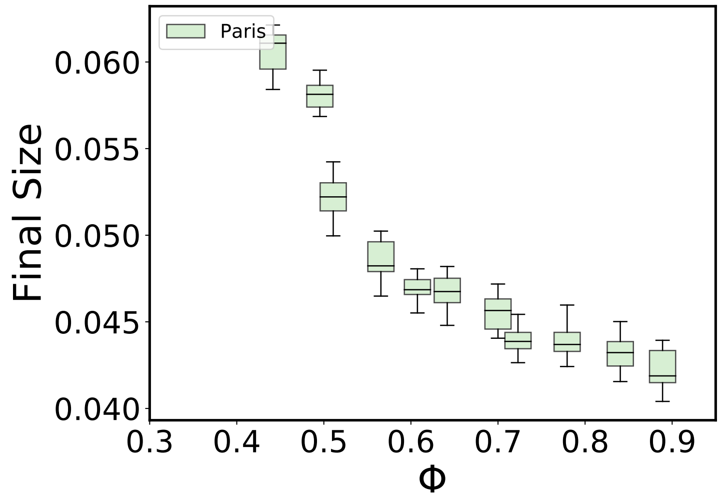

Figure S26: **Google mobility data and population according to flow: Hard lockdown** Simulations run with realistic implementation in Paris. The population is distributed proportionally to the flow of every node, as in main text. The age diversification is included following the public census data proportions. The epidemic size is explored as a function of Φ with a hard lockdown situation as described in [2] .We show that, if the mobility and social restrictions are hard enough, it is possible to erase the Φ dependence. Therefore, this crucial finding is independent of the level of detail of the model

5.4 Model with heterogeneous X_S

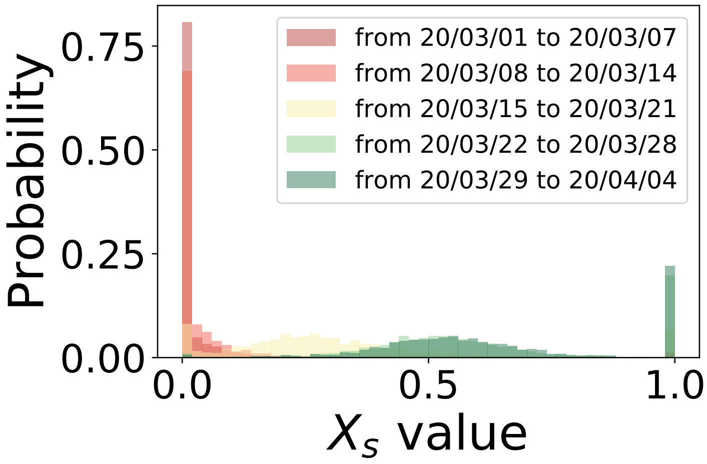

Figure S27: **Time-dependent X_S distribution: soft lockdown** Every node has its own X_S value extracted as the mobility reductions in the New York metropolitan area. The mobility could increase locally during lockdown, in these cases, $X_S = 0$. This spatial distribution is updated every week according to data.

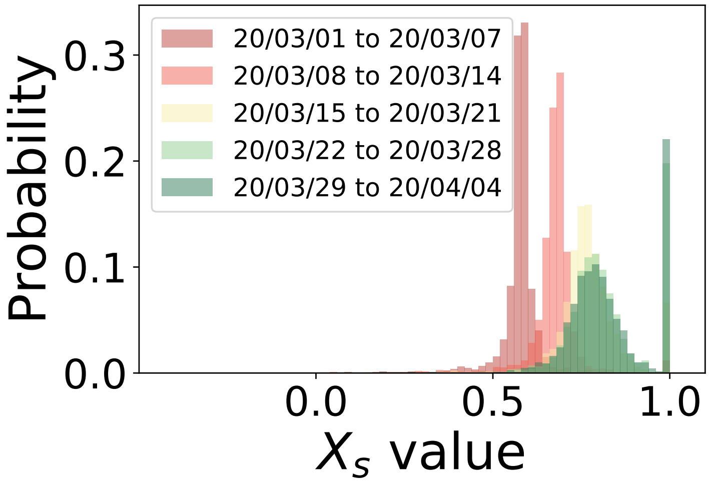

Figure S28: **Time-dependent X_S distribution: hard lockdown** Every node has its own X_S value extracted as a linear map of the mobility reduction in the New York metropolitan area. In particular we map the minimum mobility reduction to $X_S = 1$. This spatial distribution is updated every week according to data.

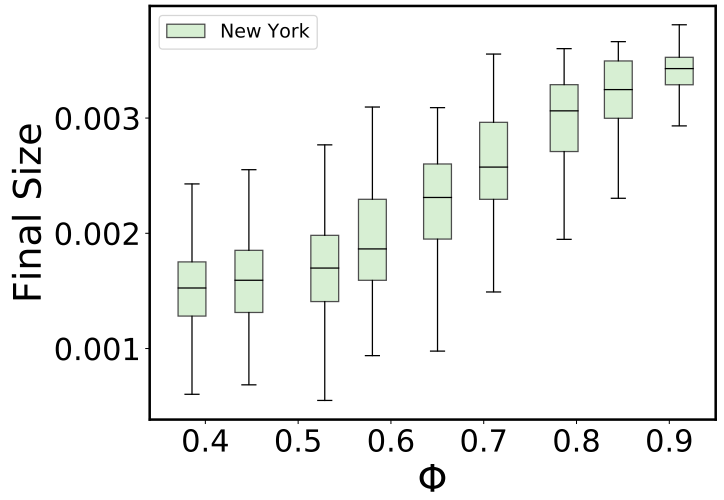

Figure S29: **Epidemic size with heterogeneous X_S: soft lockdown** Simulations run with the same parameters as Fig. 5**a** for a city generated with the mobility network and total population of the New York metropolitan area. The epidemic size after 14 weeks is explored as a function of Φ with X_S heterogeneous in space and time as in Fig. S27 . In this way, it is possible to observe that the trend showed in the Fig. 5**b** holds in the case of a temporal and spacial heterogeneous response

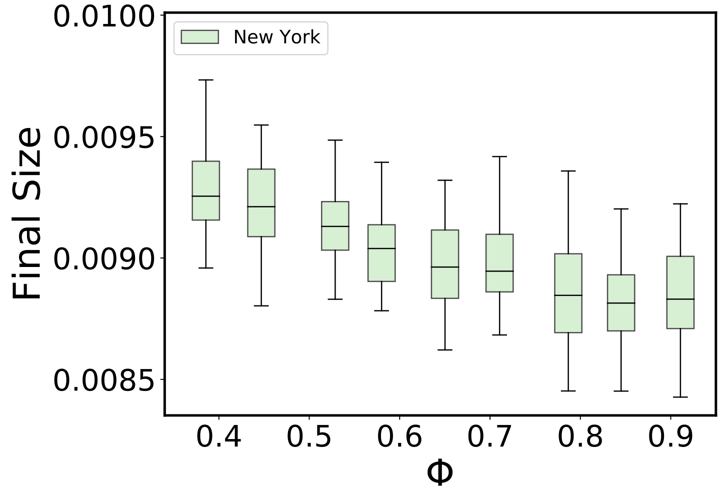

Figure S30: **Epidemic size with heterogeneous X_S: hard lockdown** Simulations run with the same parameters as Fig. 5**a** for a city generated with the mobility network and total population of the New York metropolitan area. The epidemic size is explored as a function of Φ with X_S heterogeneous in space and time as in Fig. S28 . In this way, it is possible to observe that the trend showed in the Fig. 5**a** holds in the case of a temporal and spacial heterogeneous response

5.5 Model synchronization of mobility reduction and epidemics

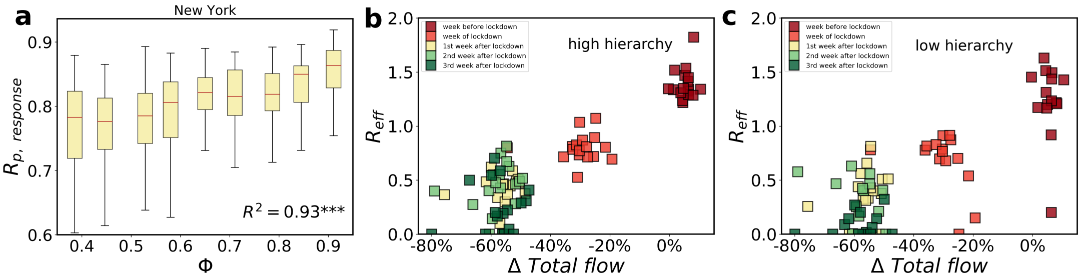

Figure S31: **Model synchronization of mobility reduction and epidemics** Synchronization of mobility reduction and contagion spread among city counties measured through the Pearson coefficient of plots as those in Fig. 1 **g-i** for New York. We take the same empirical heterogeneous mobility reduction in NY counties and rewire the city flows to produce different hierarchies. The way in which the counties epidemics synchronize reflects the hierarchy of each scenario, consistently with what we observed empirically in Fig. 3.

6 Further insights on the available COVID data and parameters estimation

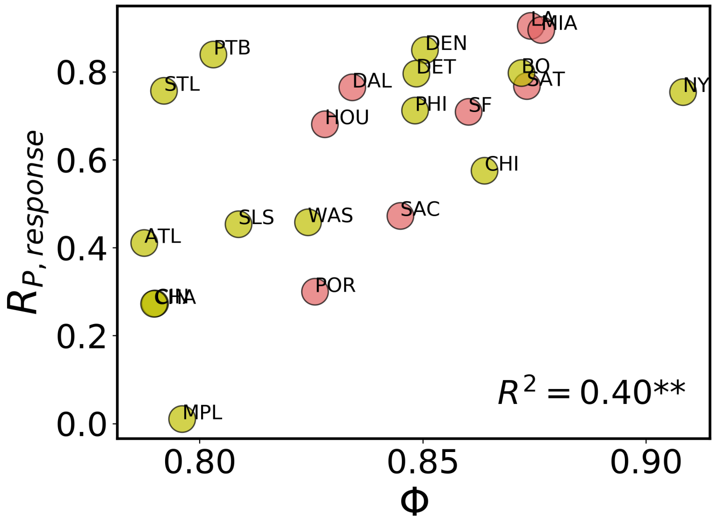

Figure S32: **Synchronization of mobility and epidemics using two weeks delay** Synchronization of mobility reduction and contagion spread among city counties measured through the Pearson coefficient of plots as those shown in Fig. 2g-i. Response to mitigation is more sensitive in cities with higher Φ. The delay between county level mobility reductions and the associated reported cases is increased to two weeks to take into consideration possible reporting delays.

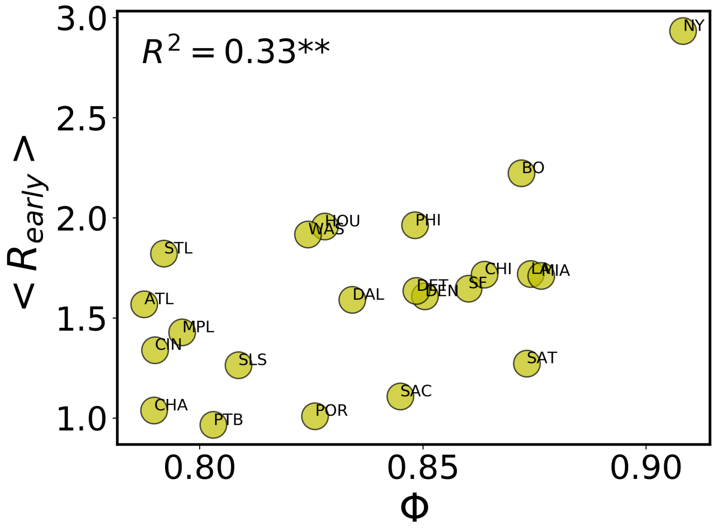

Figure S33: **Early stage estimation of R_0 one week later.** Average R_{eff} over three weeks after the first week of onset of 100 cumulative cases per city as a function of Φ. Initial transmission increases with centralization. Note that in the main paper Fig.3a we considered week 1,2 and 3 after the onset, here we consider week 2,3 and 4 in order to estimate the early stage R_{eff} with higher statistics. We do so in order to minimize the low statistics issue affecting the estimation of R_{eff} in the early stage.

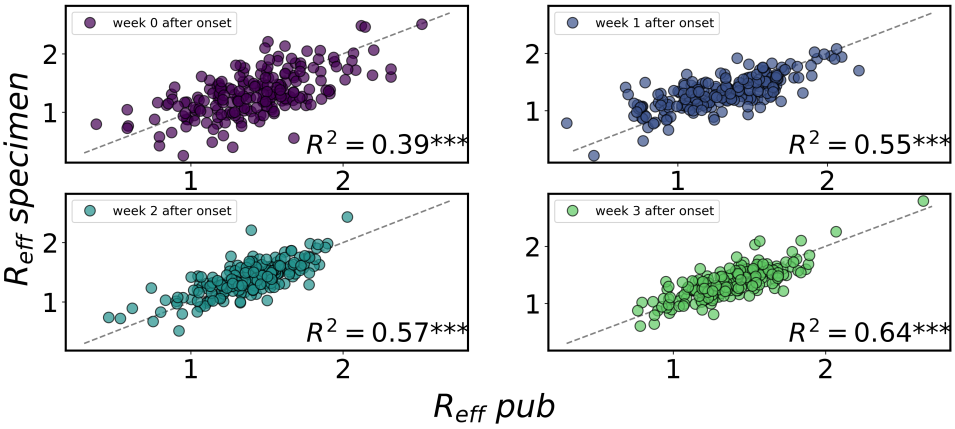

Figure S34: **Estimation of R_{eff} with different policies of cases reporting dates.** Scatter plot of comparison between R_{eff} computed on Covid19 cases by specimen date versus cases by publication date in the first 4 weeks after the onset for 210 Upper Tier Local Authorities in UK (UTLA). The difference in the estimation of R_{eff} is clearly visible in the first week, even though the correlation is good and significant, while in the following weeks the differences tend to vanish very quickly, leading to even higher correlations.

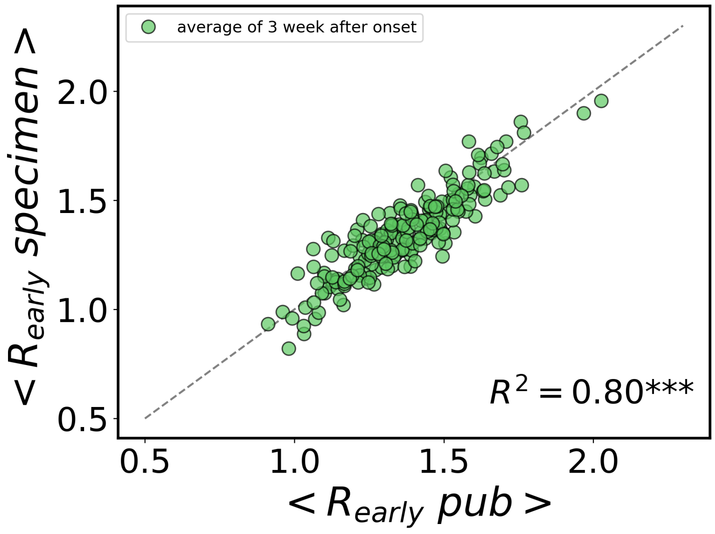

Figure S35: **Estimation of the early stage R_0 with different policies of cases reporting dates.** Scatter plot of comparison between the average R_{eff} over the first three weeks after the onset computed on the specimen date cases versus the R_{eff} computed on the publication date cases. The fluctuations registered in the first week are well compensated by the following two weeks estimations, leading to an almost coincident estimation of R_{eff}. $p_{value} = 10^{-71}$

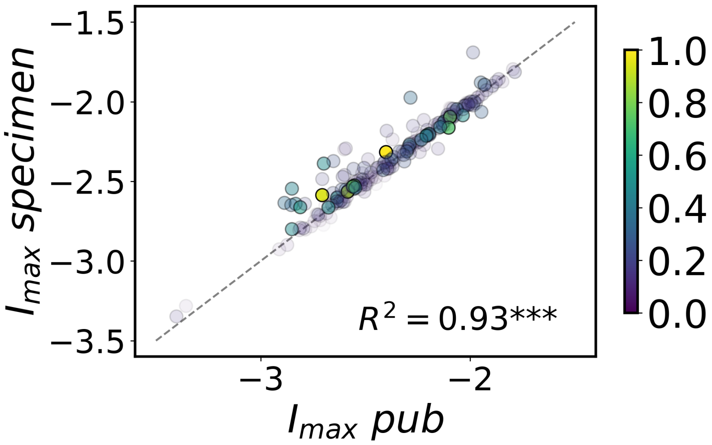

Figure S36: **Estimation of I_{max} with different policies of cases reporting dates.** Scatter plot in Log10-Log10 scale of the incidence peaks registered in 210 UK Upper Tier Local Authorities by specimen date versus cases by publication date. The peaks are defined as the sum of one week cases over the local population. Transparency and colormap are applied proportionally to the local population in a normalized fashion.

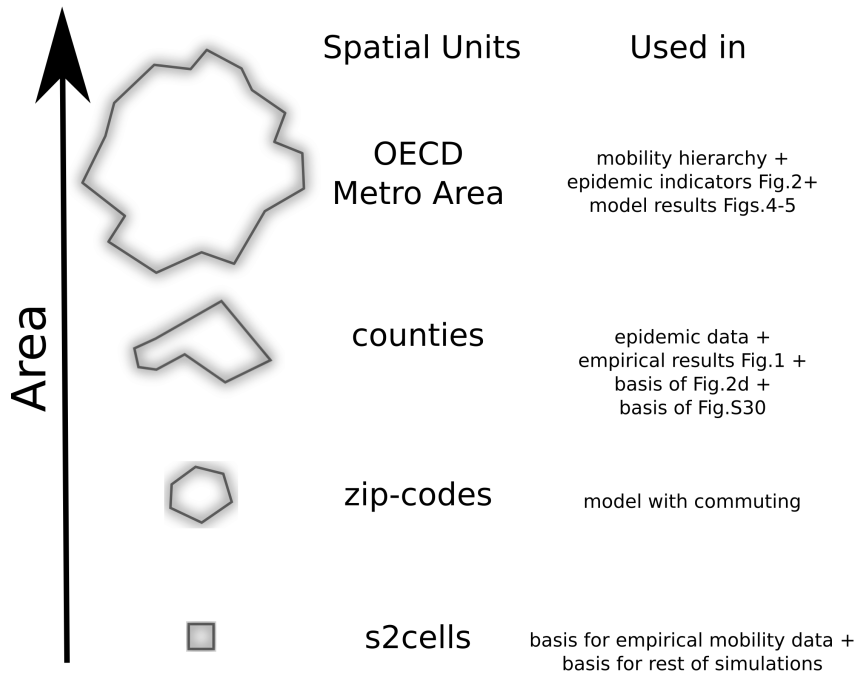

Figure S37: **Sketch figure showing the size of considered area units.** Spatial subdivisions involved in the study.

7 Variables and parameters

In this section , we offer a summary with the main variables and parameters that were introduced in the main text.

$\beta = 0.48$ days^{-1}	Infection rate$\left(S + I \xrightarrow{\beta} E + I\right)$.
$t_I = 3.8$ days	Average infectious time$\left(I \xrightarrow{t_I^{-1}} R\right)$.
$t_E = 3.7$ days	Average exposed time$\left(E \xrightarrow{t_E^{-1}} I\right)$.
$M = 0.4$	Portion of inhabitants going outside their home cell before lockdown.
$M'_i = 0.4\frac{\sum_j T_{ij}}{\sum_j T'_{ij}}$	Portion of inhabitants going outside their home cell during lockdown.
X_S	Portion of non-interacting susceptible individuals during lockdown.
π_{th}	Prevalence threshold that activates mobility restrictions.
$\tau = 1/3$ days	Average time outside residence cell.

Table S24: Epidemic model parameters

Φ	Percentage of randomized links
0.91	0% (genuine Φ value)
0.84	10%
0.78	20%
0.71	30%
0.65	40%
0.58	50%
0.52	60%
0.44	70%
0.38	80%
0.31	90%
0.25	100%

Table S25: Example on NY of the effect of the randomization procedure on Φ. As the percentage of random edges grow, the structure of the city gets lost resulting in a lower value of Φ.

- $\mathbf{T}$: Origin-destination matrix (before lockdown). Its elements (T_{ij}) encode the trip flow from the cell i to the cell j. It is extracted directly from data of a week that is representative of the mobility before the mobility restrictions were established.
- $\mathbf{T}'$: Origin-destination matrix (during lockdown). Same definition as $\mathbf{T}$, but is representative of the mobility under mobility restrictions.
- $T = \sum_{i,j} T_{ij}$: The total flow of a territory. This is, the sum of the trips over all the S2 cells present in the city
- L_i: Hotspot level of cell i (see [3] for details).
- $S_{\ell m} = \frac{\sum_{i<j} T_{ij}\,\delta(L_i,\ell)\,\delta(L_j,m)}{\sum_{i<j} T_{ij}}$: Normalized mobility matrix between hotspot levels (see [3] for details)
- $\Phi = \sum_{\ell,m} S_{\ell m}\,\{\delta(\ell,m) + \delta(\ell,m-1) + \delta(\ell-1,m)\}$: The flow hierarchy. This is the main control parameter of the work, encodes information about the topology and mobility within the cities (see [3] for details).
- $I_i(t)$: Number of new infections at cell i on day t.
- $I_{\max} = \max_t\{\sum_i I_i(t)\}$: Absolute maximum of the incidence curve.

- R_{eff}: Reproduction number measured from time series of incidence. This has been done using the methodology developed in [4] and the code available at [5].
- R_{early}: Average over three weeks of R_{early} measured at the early epidemic stages (after the city incidence has gone over a certain case onset: 100 for the empirical data and 10,000 for the models where there is no detection problem due to asymptomatic infections).
- $t(I_{\max})$: Time (day) at which the maximum of the incidence occur.
- Final size: Total portion of the population affected by the disease at the end of the outbreak.

8 Implementation of recurrent mobility in the model

Seeking for a self-contained work we summarize the main characteristics of the GLEaM algorithm used to implement the epidemic model. We refer to [6] for further details. The basic idea is to use an effective force of infection that encodes the information of the recurrent mobility ($\frac{\beta I}{N} \to \lambda(\beta, \mathbf{T}, I, S)$). The force of infection is defined so that the probability that each susceptible individual of cell j gets infected must be proportional to $\lambda_j \Delta t$. Healthy individuals can get the disease in their residence cells or while commuting. Therefore, the force of infection will have two contributions, λ_{jj} accounting for infections in the residence cell j and λ_{ji} for infections while commuting. The label i in λ_{ji} belongs to the set of neighbours of j that we note as $v(j)$. These contributions to the force of infection will still resemble the mass action principle

$$\lambda_{ji} = \frac{\beta I_i^*}{N_j^*} \frac{S_{ji}^*}{S_i}. \tag{1}$$

Where the $*$ symbol reads "effective". This is, I_j^* is the effective number of infected individuals in cell j (the sum of those infected individuals living in j plus those commuting in j). In the same way, N^* is the effective total population of cell j and S_{ji}^* is the effective number of susceptible individuals of j commuting in i. The whole strategy relies in the computation of these effective quantities.

Since the disease dynamics are slower than the mobility dynamics, we can approximate the stochastic displacements through the network by its stationary average:

$$\begin{aligned} X_{ji}^* &= \frac{X_j \frac{T_{ji}}{\tau}}{1 + \frac{\sum_{l \in v(j)} T_{jl}}{\tau}} \\ X_{jj}^* &= \frac{X_j}{1 + \frac{T_{jj}}{\tau}} \\ X_j^* &= X_{jj}^* + \sum_{i \in v(j)} X_{ji}^* \end{aligned} \tag{2}$$

Where X can be any of the compartments I and S and also the entire population N. Finally, λ_j is calculated as:

$$\lambda_j = \frac{\lambda_{jj}}{1 + \frac{\sum_{l \in v(j)} T_{jl}}{\tau}} + \sum_{i \in v(j)} \frac{\lambda_{ji} \frac{T_{ji}/}{\tau}}{1 + \frac{\sum_{l \in v(j)} T_{jl}}{\tau}}, \tag{3}$$

Supplementary References

[1] Bossomaier, T., Barnett, L., Harré, M., and Lizier, J. T. *An introduction to transfer entropy*, chapter 4, 65–95. Nature Springer, Gewerbestrasse 11, 6330 Cham, Switzerland (2016).

[2] Di Domenico, L., Pullano, G., Sabbatini, C. E., Boëlle, P.-Y., and Colizza, V. Impact of lockdown on COVID-19 epidemic in île-de-france and possible exit strategies. *BMC Medicine* **18**, 1–13 (2020).

[3] Bassolas, A., Barbosa-Filho, H., Dickinson, B., Dotiwalla, X., Eastham, P., Gallotti, R., Ghoshal, G., Gipson, B., Hazarie, S. A., Kautz, H., et al. Hierarchical organization of urban mobility and its connection with city livability. *Nature Communications* **10**, 4817 (2019).

[4] Bettencourt, L. M. and Ribeiro, R. M. Real time bayesian estimation of the epidemic potential of emerging infectious diseases. *PLOS ONE* **3** (2008).

[5] Tizzoni, M. Estimating covid-19's rt in real-time, (2020).

[6] Balcan, D., Gonçalves, B., Hu, H., Ramasco, J. J., Colizza, V., and Vespignani, A. Modeling the spatial spread of infectious diseases: The global epidemic and mobility computational model. *Journal of Computational Science* **1**, 132–145 (2010).
